# Early versus Late Surgical Treatment for Neurogenic Thoracic Outlet Syndrome

**DOI:** 10.1155/2013/673020

**Published:** 2013-09-10

**Authors:** Jasem Yousef Al-Hashel, Ashraf Ali M. A. El Shorbgy, Samar Farouk Ahmed, Rawhia R. Elshereef

**Affiliations:** ^1^Department of Neurology, Ibn Sina Hospital, P.O. Box 25427, Safat 13115, Kuwait; ^2^Department of Medicine, Kuwait University, P.O. Box 24923, Safat 13110, Kuwait; ^3^Department of Cardiothoracic Surgery, Minia University, P.O. Box 61519, Minia 61111, Egypt; ^4^Department of Neurology and Psychiatry, Minia University, P.O. Box 61519, Minia 61111, Egypt; ^5^Department of Rheumatology, Minia University, P.O. Box 61519, Minia 61111, Egypt

## Abstract

*Objectives*. To compare the outcome of early surgical intervention versus late surgical treatment in cases of neurogenic thoracic outlet syndrome (NTOS). *Design*. Prospective study. *Settings*. Secondary care (Al-Minia University Hospital, Egypt) from 2007 to 2010. *Participants*. Thirty-five patients of NTOS (25 women and 10 men, aged 20–52 years), were classified into 2 groups. First group (20 patients) was operated within 3 months of the onset and the second group (15 patients) was operated 6 months after physiotherapy. *Interventions*. All patients were operated via supraclavicular surgical approach. *Outcomes Measures*. Both groups were evaluated clinically and, neurophysiologically and answered the disabilities of the arm, shoulder, and hand (DASH) questionnaire preoperatively and 6 months after the surgery. *Results*. Paraesthesia, pain, and sensory nerve action potential (SNAP) of ulnar nerve were significantly improved in group one. Muscle weakness and denervation in electromyography EMG were less frequent in group one. The postoperative DASH score improved in both groups but it was less significant in group two (*P* < .001 in group 1 and *P* < .05 in group 2). *Conclusions*. Surgical treatment of NTOS improves functional disability and stop degeneration of the nerves. Early surgical treatment decreases the occurrence of muscle wasting and denervation of nerves compared to late surgery.

## 1. Introduction

Thoracic outlet syndrome (TOS) is defined as a group of clinical symptoms caused by the entrapment of neurovascular structures (subclavian vessels and the brachial plexus) en route to the upper limb via the superior thoracic outlet [1]. This is generally due to a congenital bony anomaly either because of the presence of a cervical rib, a prolongation of the C7 transverse process, or being secondary to fibrous bands or anomalous muscles [2]. Also, trauma such as hyperextension-flexion injuries of arm, neck trauma due to motor vehicle accidents, repetitive stress injury, and trauma that causes chronic cervical muscle spasm may precipitate NTOS [3, 4]. Most of the patients 95% have a neurogenic form, 2% have venous symptoms and only 1% has clinical arterial compression [5]. The diagnosis of neurogenic TOS is relatively straightforward. It is presented with pain and sensory disturbance predominantly in the ulnar forearm and hand, aggravated by use of the affected limb. Weakness and wasting of the small hand muscles are also reported. Neurophysiological studies confirming chronic postganglionic axonal loss, and excluding focal mono-neuropathy [6].

Surgical management is controversial due to variation in reported success and complications and due to the good results presented in some studies that use conservative treatment [2]. The optimal time of surgery had not been studied before. The aim of our study is to compare the functional outcomes in patients who were operated on early versus those who were operated on after 6 months of medical and physical therapy. 

## 2. Patients and Methods

### 2.1. Patients

This study included 35 patients with NTOS of both sexes. They are collected from Minia Hospital University in the period from 2007 till 2010. The study excluded patients with other nerve entrapment, cervical disc disease, or traumatic TOS. The included patients were classified into 2 groups. The first group of 15 patients who underwent medical and physical therapy for six months (include modalities which improve pain (interferential, ultrasound, TENS, massage, and sometimes LASER) with exercise of the different muscular groups. This consisted of pectoralis stretching, strengthening the muscles between the shoulder blades, good posture, and active neck exercises (including chin tuck, flexion, rotation, lateral bending, and circumduction). Patients who had no satisfactory improvement were operated on. The other group of 20 patients was operated on within 3 months of the onset of NTOS if there is no improvement of medical and physical therapy.

 All patients signed informed consent for surgery. All patients were operated on via supraclavicular approach by one cardiothoracic surgeon for NTOS without associated vascular complications. Six patients had bilateral operations (4 women and 2 men).

### 2.2. Methods

Both groups of patients underwent the following.

- Preoperative assessment:
  1. clinical history and physical examination with provocative maneuvers for thoracic outlet syndrome (Adson, Wright, elevated arm stress, and costoclavicular tests),
  2. X-rays of the thorax and spinal column to determine possible anatomic anomalies, magnetic resonance imaging (MRI) in all patients to confirm the diagnosis, and rule out cervical disc disease,
  3. electrodiagnostic studies (EDS) are required to objectively confirm the diagnosis of NTOS and to rule out other entrapment neuropathy, polyneuropathy, and motor neuron disease, and to compare the condition before and after operation,
  4. DASH questionnaire was used for assessment of functional disability.
- Postoperative assessment (6 months after surgery):
  1. followup of presenting symptoms and signs,
  2. followup of electrodiagnostic studies (EDS),
  3. DASH questionnaire to measure clinical and functional improvement.

#### 2.2.1. Electrodiagnostic Studies (EDS Criteria) [7–10]

1. Absent or reduced amplitude (<12 mV) of the ulnar sensory nerve action potential (SNAP),
2. Absent or prolonged minimum latency (>33 msec) of the ulnar F-wave (with or without abnormalities of the median F-wave), and with normal F-waves in the contralateral (unaffected) upper extremity or
3. Needle electromyography (EMG) showing denervation (e.g., fibrillation potentials or positive sharp waves) in at least one muscle supplied by each of two different nerves from the lower trunk of the brachial plexus,
4. Normal amplitude (≥15 mV) of the median nerve SNAP and
5. Normal conduction velocity (≥50 m/s) of the ulnar motor nerve across the elbow.

#### 2.2.2. Provocative Tests [11, 12]

Include the following. 

*Elevated Arm Stress Test (EAST or Roos Test)*. The patient places the affected arm in full abduction and external rotation and then opens and closes the hands slowly for 3 minutes. This test constricts the costoclavicular space. It is considered abnormal if typical symptoms are elicited and the patient cannot sustain this activity for the full 3 minutes.

*Adson Test*. The patient extends the neck and rotates the head toward the involved extremity, which is held extended at the side. This test constricts the interscalene triangle. It is considered abnormal if a change in the radial pulse is detected when the patient inhales deeply and holds their breath. 

*Wright Test*. The patient sits or stands with the arm in full abduction and external rotation. This test constricts the costoclavicular space. It is considered abnormal if typical symptoms are elicited and a change in pulse is detected. 

*Costoclavicular Test*. The examiner depresses the patient's shoulder. This test constricts the costoclavicular space and creates tension across the pectoralis minor. It is considered abnormal if typical symptoms are elicited.

#### 2.2.3. The DASH Questionnaire

It was developed by the American Academy of Traumatology, the Institute of Work and Health, and the Council for the Society of American muscular-skeletal specialities (http://www.dash.iwh.on.ca/) [2]. The questionnaire includes 30 questions related to symptoms of daily activities and social and psychological functions. Scores range from 0 points (asymptomatic) to 100 (totally invalid). The score was calculated for each patient using the algorithm: ([sum of responses/*n*] − 1) × 25, where *n* = number of completed responses.

#### 2.2.4. Operative Technique

All patients were operated on using general anesthesia with endotracheal intubation. As in supine position a big roll was placed under the cervical and thoracic spine, and the head was turned in the opposite direction. A supraclavicular incision is made one fingerbreadth above the clavicle extending from 5 to 7 cm. The platysma is divided and subplatysma flaps are created. The omohyoid muscle is divided and two to three cm are resected to avoid a loose muscle end adhering to nerve roots postoperatively and causing recurrent symptoms. The scalene fat pad is dissected and retracted laterally avoiding thoracic duct injury in the left side. The brachial plexus was exposed in the usual fashion using a traumatic techniques [13]. The phrenic nerve is identified on the anterior scalene muscle. It usually runs from lateral to medial as it descends toward the clavicle as it lies just below the transverse cervical artery. Vessel loop is used to retract and protect it from the anterior scalene muscle during its dissection and division from its first rib insertion. The anterior scalene muscle mobilized cranially over 3-4 cm; a segment of the muscle was excised especially if the 1st rib is not excised to avoid reinsertion and adhesions. The first rib was carefully exposed (primary TOS), and the periosteum and muscle tissue were elevated off the rib while the pleura was retracted posteriorly and inferiorly using a blunt retractor. Then, the plexus was carefully identified and retracted. The middle scalene muscle excision begins by gently identifying and retracting the long thoracic nerve, excision moving from lateral to medial, taking the insertion off the first rib and excising the cephalic end as high as is safe. When present, a cervical rib lies in the midst of the middle scalene muscle. Using Shumacker rib cutter, cervical rib is cut in the middle and, then, by rocking movement, dislocated from the C7 and 1st rib, raney bone rongeur is used to excise any remnants, when needed the first rib was transected anteriorly and posteriorly, and then retraction on the rib, intercostal muscle fibers, and scalene muscle fibers were removed to permit complete removal of an approximately 8–10 cm segment of the first rib. The phrenic nerve and subclavian artery must be observed, mobilized, and encircled with vessel loops to avoid the rib cutter as it encircles the rib. The subclavian vein is protected with a thin, long retractor over it. The anesthesiologist puts the patient in exhalation to reduce chances of entering the pleura. Utilizing bone Rongeurs, as much of the anterior and posterior remnants of the first rib were removed leaving a wide open space below the clavicle. Prior to removing the 1st rib, intraoperative abduction and external rotation maneuver of the upper extremity brought the clavicle inferiorly and posteriorly which reveal any 1st rib compression of the vascular and neural elements. Following the first rib removal, executing the same maneuver of abduction and external rotation, it was certain that there was no residual compression of the vascular and neural structures. Following the first rib resection, no neurolysis of the brachial plexus was performed. Haemostasis, instillation of solumedrol and testing for pleural and thoracic duct injury before closure of in layers A were done. 10 French round suction drain is inserted below the plexus and brought out through the lateral corner of the incision. All the patients were operated on under general anesthesia, via supraclavicular approach by single cardiothoracic surgeon. The anterior scalene muscle was sectioned and 3-4 cm segment is excised in all the cases preserving the nervous structures. The cervical rib was resected in 20 patients, the first rib was resected in 10 patients (2 cases with large anomalous first thoracic rib and 8 cases indicated when intraoperative abduction and external rotation maneuver of the upper extremity brought the clavicle inferiorly and posteriorly with severe compression of the vascular and neural elements). An elongated C7 transverse process is punched and the stump is smoothed with the Raney rongeur in 3 cases. The postoperative complications were collected.

Data were analyzed by the Statistical Package for the Social Sciences (SPSS, version 11.0 under windows) [14]. Simple descriptive statistical tests (mean and standard deviation) are used to describe the numerical values of the sample. Paired-sample *t* test was applied to compare the parametric variables between two subgroups. Chi-square test “*χ* ^2^” was applied to compare frequencies and proportions. A probability of (*P*)≤.05 is accepted as significant.

## 3. Results

Thirty-five patients who had been prospectively followed 6 months after surgical treatment for NTOS including 25 women (71.4%) and 10 men (28.6%) with a mean age of 34.51 ± 8.6 years. The age of patients ranged from 20 to 52 years. After radiological studies cervical rib was diagnosed in twenty-three patients (65.75%), an elongated C7 transverse process in 10 patients (28.6%), and a large anomalous first thoracic rib in two patients (5.7%). Patients were classified into 2 groups. Surgery was reserved for first group (15 patients) with a clinical diagnosis of NTOS who had failed to improve according to patient satisfaction in terms of pain control and improvement in work and daily activities after six months of physical therapy. The second group (20 patients) underwent surgery within 3 months of the diagnosis and conservative treatment.

### 3.1. Preoperative Assessment of Patients 

Showed the following results. The most frequent symptom was paraesthesia in 30 patients (85.7%) that worsened during physical exercise: eleven of them in group 1 and the other 19 in group 11. Pain was reported in 25 patients (71.4%). It was reported in 10 patients in group 1 and in 15 patients in group 11. Weakness of intrinsic hand muscles or flexor digitorum profundus was reported in 11 patients (31.4%), six of them in group 1 and the other 5 in group 2. Three patients (8.6%) presented with atrophy of the hypothenar muscles on physical examination; all of them were in group 1. Electrophysiology was abnormal in both groups. Most commonly observed, absent, or reduced amplitude of the ulnar SNAP is in 22 patients (62.9%): twelve of them in group 1 and the other 10 in group 2. EMG showed evidence of denervation of small hand muscles in 7 patients (46.4%), all from group 1. Prolonged F-wave is seen in five patients, four from group 1 and only one from group 2. Both groups answered the DASH questionnaire preoperatively (Table 1).

### 3.2. Surgical Outcome 6 Months Postoperatively

Paraesthesia and pain were significantly less reported in group 11 compared to group 1. Muscle weakness was less frequent in group 11 compared to group 1, but the difference was not significant. Muscle wasting was still reported only in group 1 SNAP of ulnar nerve and denervation in EMG showed significant improvement in group 11 compared to group 1 (Table 2).

All the patients answered the DASH questionnaire preoperatively and 6 months after the surgery. The postoperative score was significantly better in group II compared to group 1 (Table 2). The postoperative score in group 1 was 23.5 ± 13.9, and the improvement was not significant compared to the preoperative score 34.2 ± 13.3. In group II the postoperative score was 10.5 ± 5.7. The improvement was significant compared to their preoperative score 27.3 ± 10.5. The postoperative DASH score improved in both groups, but it was no significant in group 1 compared to group 11 (*P* < .07 in group 1 and *P* < .001 in group 2) (Table 3).

The complications after surgery were pneumothorax (2 cases complicating the 1st rib resection) treated with intercostal tube insertion for 24 hours, phrenic nerve palsy (1 case complicating mobilization and traction on phrenic nerve) cough stops 1 month postoperatively without surgical intervention and wound complications (2 cases of wound hematoma and Infection treated with antibiotic and proper wound care for 1 week/1 case of wound scare keloid formation treated topically by corticosteroid creams and injection). No haemothorax, no long thoracic nerve palsy, and no brachial plexus injury was noted in any patient.

## 4. Discussion

Patients who develop TOS usually are middle age and they have led an active life without symptoms until certain job activities or trauma-induced upper extremity pain, numbness, or weakness. The management of TOS can be both nonoperative and operative. Nonoperative management includes modification of behavior by avoiding provocative activities and arm positions, in addition to individually tailored physical therapy programs [15]. The indications for surgical treatment and the choice of the correct type of procedure are still a subject for discussion because of the frequency of recurrence and complications. Also, the methodology for evaluation of the results needs unification [16]. Currently, the most frequently used method for decompression of the thoracic outlet (inlet) is transaxillary first rib resection [17, 18]. However, different publications suggest that this method alone results in a recurrence rate of approximately 20–30% in experienced hands [19, 20]. Supraclavicular approach for thoracic outlet decompression is less popular than the transaxillary approach but has been advocated by several authors [21, 22].

We regard the supraclavicular approach as the operation of choice in patients with NTOS who require surgical treatment. It provides the best exposure of the neurovascular bundle, cervical ribs, and fibrous bands and can be used for the first rib resection. Its disadvantages include the risk of damage to the long thoracic or phrenic nerves and the presence of a cosmetically undesirable scar. The objectives of surgical treatment for NTOS are to diminish the symptoms and prevent irreversible damage of the brachial plexus. One cardiothoracic surgeon via supraclavicular approach is better to fix the surgical technique adopted and avoid different individual surgeon variation. We specify NTOS with narrow inclusion criteria to get the most accurate results from our work. 

In this study, early surgical treatment for neurogenic TOS has reported significant surgical outcome compared to late surgical treatment. Pain and sensory disturbance improved significantly in group 2 compared to group 1, whereas improved muscle strength was nonsignificant in group 2 compared to group 1. Muscle wasting never resolved in patients had late surgery, which suggests that early surgery may prevent irreversible denervation of hand muscles. Late surgical treatment will improve pain and sensory disturbance but will not allow a useful recovery of muscle function and merely prevents further progression of the weakness and wasting. 

In the study, also, electromyography shows denervation of forearm flexors, small hand muscles, and reduced or absent amplitude of ulnar sensory nerve action potentials, and this confirms that the underlying lesion is postganglionic. This result is in agreement with previous results [10, 23]. The patients with pathological EMG were in group one and had no significant improvement after thoracic outlet decompression. All of our patients with normal electrophysiological results were in group 2 and they showed significant benefit from surgical treatment. This result is in agreement with previous studies as well [24]. 

Functional prognosis is the main factor in determining the outcome of surgery. However most studies have failed to use an objective measure of outcome [2]. We used the DASH questionnaire as its questions encompass a very broad clinical spectrum. Early surgical treatment for the patients with neurogenic TOS showed a highly significant improvement in their DASH scale compared to improvement of patients with late surgical treatment who also improved, but less significant than in patients with early surgical intervention. The result of postoperative improvement in DASH questionnaire is similar to the result of other authors [4], who reported that DASH scores, improved (*P* < .001) for their neurogenic patients. The more significant improvement in group 2 compared to group 1 could be explained by the presence of patients with muscle wasting and the presence of pathological EMG only in group 1. These results are in agreement with previous studies [15, 23, 24] who reported that lack of improvement after surgery in their patients (according to DASH) was caused by irreversible degeneration of the brachial plexus shown in electrophysiological examination. So, the patients with normal or minimal neurophysiological studies who otherwise meet the criteria for the diagnosis of TOS should not be excluded from being considered for surgical treatment if other indications are convincing. 

We recorded surgical complications in 5 out of 35 patients but these did not give rise to long-term or permanent symptoms or disability.

## 5. Conclusion

Early surgical intervention for NTOS prevents the degeneration of the brachial plexus and leads to better functional outcome than delayed treatment. Physiotherapy is more effective after early surgical intervention than before and late surgical intervention. Also, when the surgery was done late, it will improve some of the symptoms but the denervation will not resolve.

## Figures and Tables

**Table 1:** Preoperative assessment of both groups.

Clinical presentation	Group I (late surgical treatment) *n* = 15	Group II (early surgical treatment) *n* = 20
Paraesthesia	11 (73.7%)	19 (95%)
Pain	10 (66.7%)	15 (75%)
Muscle weakness	6 (40%)	5 (25%)
Muscle wasting	3 (20%)	0
Positive provocative tests	6 (40%)	12 (60%)
Reduced or absent SNAP of ulnar nerve	12 (80%)	10 (50%)
Denervation in EMG	7 (46.7%)	0
Prolonged F latency	4 (26.7%)	1 (5%)
DASH	34.2 ± 13.3	27.3 ± 10.5

**Table 2:** Comparisons of function outcome postoperative in both groups.

Clinical presentation	Group I (*n* = 15)	Group II (*n* = 20)	*P*
Paraesthesia	5 (33.3%)	2 (10%)	.05*
Pain	4 (26.7%)	1 (5%)	.05*
Muscle weakness	3 (20%)	1 (5%)	.06
Muscle wasting	3 (20%)	0	.07
Reduced SNAP of ulnar nerve	6 (40%)	0	.003**
Denervation in EMG	5 (33.3%)	0	.009**
Prolonged F latency	2 (13.35%)	0	.176
DASH score	29.5 ± 13.9	10.5 ± 5.7	.001**

*Significant, **highly significant.

**Table 3:** Surgical outcome of functional disability 6 months postoperatively.

DASH score	Preoperative	Postoperative	*P*
Group I (*n* = 15)	34.2 ± 13.3	23.5 ± 13.9	.07
Group II (*n* = 20)	27.3 ± 10.5	10.5 ± 5.7	.001**

*Significant, **highly significant.

## List of Abbreviations

DASH: “Disabilities of the Arm, Shoulder and Hand”
NTOS: Neurogenic thoracic outlet syndrome
SNAP: Sensory nerve action potential
EMG: Electromyography.
